# Emergency Department Response to SARS, Taiwan

**DOI:** 10.3201/eid1107.040917

**Published:** 2005-07

**Authors:** Wei-Kung Chen, Hong-Dar Isaac Wu, Cheng-Chieh Lin, Yi-Chang Cheng

**Affiliations:** * China Medical University Hospital, Taichung, Taiwan;; † China Medical University, Taichung, Taiwan

**Keywords:** epidemic, infection control, outbreak, severe acute respiratory syndrome, Taiwan, emergency services, hospitals

## Abstract

How emergency departments of different levels and types cope with a large-scale contagious infectious disease is unclear. We retrospectively analyzed the response of 100 emergency departments regarding use of personal protective equipment (PPE) and implementation of infection control measures (ICMs) during the severe acute respiratory syndrome outbreak in Taiwan. Emergency department workers in large hospitals were more severely affected by the epidemic. Large hospitals or public hospitals were more likely to use respirators. Small hospitals implemented more restrictive ICMs. Most emergency departments provided PPE (80%) and implemented ICMs (66%) at late stages of the outbreak. Instructions to use PPE or ICMs more frequently originated by emergency department administrators. The difficulty of implementing ICMs was significantly negatively correlated with their effectiveness. Because ability to prepare for and respond to emerging infectious diseases varies among hospitals, grouping infectious patients in a centralized location in an early stage of infection may reduce the extent of epidemics.

Severe acute respiratory syndrome (SARS) is a newly emerging infectious disease in humans. The initial outbreak, which occurred in November 2002 in China, marked the beginning of a pandemic that spread rapidly around the globe, resulting in >8,000 reported cases (1). Taiwan was the third region to be affected by the outbreak because of its frequent contact with China and Hong Kong. The first case in Taiwan was reported on February 1, 2003, and a total of 671 probable cases had been reported by June 15 (2). The turning point of the SARS outbreak in Taiwan occurred when a healthcare laundry worker with atypical SARS symptoms visited the emergency department of hospital A (located at Taipei City) 3 times (on April 12, 14, and 15) and was admitted to an ordinary ward without quarantine (3). The outbreak at hospital A occurred on April 22, then spread from hospital A to other hospitals. Before the hospital A outbreak, most hospitals did not anticipate the extent of the SARS epidemic.

Many healthcare workers (HCWs) were infected during the SARS epidemic (3–8). Protecting HCWs from contamination was the first priority of infection control measures (ICMs) in hospitals. In response to the growing epidemic, most hospitals followed the recommendations of the Department of Health (DOH), which were similar to those of the Centers for Disease Control and Prevention and the World Health Organization (9–11). However, in some hospitals, additional measures were also taken to prevent a hospital outbreak when hospital managers believed that the existing recommendations were ineffective or insufficient. During the SARS outbreak, the emergency department played a vital role in infection control because many patients with fever sought medical attention in an emergency department. Facing a new disease, emergency department personnel were unable to make decisions regarding timing of personal protective equipment (PPE) usage and classification of infectious disease because the means of transmission were unclear and early identification was difficult (unclear clinical symptoms and lack of a laboratory test).

Previous studies have demonstrated that effectively implementing ICMs can control and prevent an outbreak (12,13). However, the problem is not just a question of control in individual hospitals. The control measures must be coordinated throughout the healthcare system, and these measures must be implemented in the initial stage, not just in the late stage. Whether emergency departments had the ability to make adequate preparations or implement all the necessary ICMs was unclear. Because policymakers lacked adequate information about the capacity and ability of hospital or emergency departments to implement such measures, no decisions were made in the early stages of the outbreak about whether to divert or group persons with suspected or probable cases of SARS into a centralized section in emergency departments. The purpose of this hospital-based study was to collect data from hospitals of various levels and analyze the ability of the hospitals' emergency departments to cope with the SARS outbreak. These data may be used to improve the effectiveness of existing emergency protocols before the reemergence of SARS, influenza, or other infectious disease. Hence, policymakers, as well as administrators of hospitals and emergency departments, will be able to make more effective decisions in the early stage of an infectious disease.

## Materials and Methods

Taiwan had 468 hospitals in 2003, 393 (83.9%) private and 75 (16.1%) public. Data were collected from emergency departments in which the number of patients exceeded 500 per month. Questionnaires were sent to chiefs of staff at emergency departments in late June 2003. The questionnaire was designed by emergency department experts after panel discussions. The data collected included the following: accreditation of the hospitals, the average monthly volume of emergency department patients from March to May 2003, the effects of the SARS epidemic on emergency department workers, types of PPE supplied, kinds of ICMs implemented, as well as timing and origin of instruction to use PPE and ICMs during the SARS epidemic.

Before data analyses, hospitals were classified into 3 levels, medical center (level A), regional hospital (level B), or local hospital (level C). In general, the number of hospital beds at these hospitals, based on reference data, was >500 in level A, 200–500 in level B, and <200 in level C. The average monthly volume of emergency department patients was an average of the monthly emergency department volumes during the 3-month period. The hospitals were grouped first by level, and then the average was calculated. The average of the averages for each hospital was then determined. Emergency department workers were classified as physicians, nursing staff, or paramedics. The effects on emergency department staff were measured according to a 4-point scale, as follows: 1) had fever and needed to stay at home, 2) quarantined at home with fever or no fever, 3) quarantined at hospital, and 4) probably had SARS.

The basic PPE recommended by the DOH included head and shoe covers, goggles, face shield, gloves, apron, disposable gown, surgical mask, and N95 respiratory mask. Hand hygiene was excluded as a protection measure because accurate assessment was difficult. The high-level protective respirators were defined as P100/ N100/ FFP3 (approved by the National Institute for Occupational Safety and Health [NIOSH]) and powered air-purifying respirators with full-body isolation suit. ICMs included the guidelines from the DOH (defined as basic ICMs) as well as additional measures, such as having a fever triage ward or referral to a SARS screening team, implemented by emergency departments during the SARS outbreak. The timing of complete PPE implementation or having ICMs completely in place was classified into 2 stages: 1) early stage, from March to late April, 2) late stage, from late April to mid-June. The order to use PPE or ICMs came from 3 sources: 1) emergency department workers themselves, 2) emergency department administrators, and 3) hospital administrators. The difficulty of implementing or instituting ICMs was rated on a scale from 1 (mildly difficult) to 5 (very difficult). The effectiveness of implementing ICMs was rated on a scale from 1 (less effective) to 5 (very effective). All ratings were based on self-assessments of hospital staff.

One-way analysis of variance (ANOVA) was used to test the differences in the average monthly volumes of patients among different levels and types of emergency departments. The associations between categorical variables were analyzed by chi-square and Fisher exact test. The rating scale of difficulty and effectiveness of ICMs was represented by median and interquartile range (IQR). The correlation between difficulty and effectiveness was analyzed by Spearman rank correlation; p values <0.05 were considered significant.

## Results

A total of 213 emergency departments were initially included in this study; 152 (71.4%) were private hospitals and 61 (28.6%) were public hospitals. One hundred emergency departments responded to the questionnaire (respondent rate = 46.9%). Among these, 15 emergency departments were medical centers (respondent rate = 65.2%, including 6 public and 9 private emergency departments), 28 emergency departments were regional hospitals (respondent rate = 38.9%, including 10 public and 18 private), and 57 emergency departments (respondent rate = 44.9%, including 14 public and 43 private) were local hospitals. The overall response rate was 46.0% in public hospitals and 49.1% in private hospitals.

The emergency department volumes and assessment of the effects of the SARS outbreak on emergency department workers are shown in Table 1. From March to May 2003, the average monthly volume of emergency department patients in level A hospitals was 6,200 (range 3,429–11,080) and 3,828 (range 1,864–5,770) in level B hospitals, both of which were significantly larger than the average number of patients in level C hospitals (average 2,246, range 729–3,236) (p = 0.001). No significant differences in emergency department volume were found between public (average 2,642, range 1,364–6,258) and private hospitals (average 3,398, range 729–11,080). The most frequent effect of the SARS outbreak on emergency department workers was "fever and needed to stay at home." Emergency department workers in level A and B hospitals had a higher probability of being affected during the SARS outbreak, regardless of job type. When the effects of the SARS outbreak on public and private hospitals were compared, significant differences were found between the type of quarantine at hospitals.

**Table 1:** Effects of SARS epidemic on emergency departments*

No. EDs with ED staff affected as follows†	Hospital level				Hospital type		
	A (%), N = 15	B (%), N = 28	C (%), N = 57	p value	Public (%), N = 30	Private (%), N = 70	p value
Fever and needed to stay at home	7 (47)	8 (29)	5 (9)	0.002	7 (23)	13 (19)	0.585
Physician	6 (40)	1 (4)	0	0.000	2 (7)	5 (7)	0.932
Nursing staff	6 (40)	7 (25)	5 (9)	0.010	7 (23)	11 (16)	0.363
Paramedic	2 (13)	4 (14)	0	0.014	2 (7)	4 (6)	0.854
Quarantine at home	3 (20)	7 (25)	6 (11)	0.208	7 (23)	9 (13)	0.190
Physician	3 (20)	6(21)	4 (7)	0.122	7 (23)	6 (9)	0.044
Nursing staff	3 (20)	6 (21)	4 (7)	0.122	5 (17)	8 (11)	0.475
Paramedic	2 (13)	3 (11)	3 (5)	0.487	3 (10)	5 (7)	0.629
Quarantine in hospital	4 (27)	2 (7)	0	0.001	5 (17)	1 (1)	0.003
Physician	3 (20)	1 (4)	0	0.002	4 (13)	0	0.002
Nursing staff	3 (20)	2 (7)	0	0.006	4 (13)	1 (1)	0.012
Paramedic	3 (20)	1 (4)	0	0.002	3 (10)	1 (1)	0.045
Probable case-patients	3 (20)	3 (11)	0	0.007	4 (13)	2 (3)	0.043
Physician	1 (7)	0	1(2)	0.057	0	1 (1)	0.511
Nursing staff	3 (20)	2 (7)	0	0.006	3 (10)	2 (3)	0.133
Paramedic	3 (20)	2 (7)	0	0.006	3 (10)	2 (3)	0.133

*SARS, severe acute respiratory syndrome; ED, emergency department. †Represents the number of EDs that responded "yes" to at least 1 ED staff member in a given category of possible SARS impact. The number in parentheses is the percentage of the total EDs in a particular hospital level or type.

PPE supplied by emergency departments is shown in Table 2. The use of basic PPE did not differ significantly among emergency departments at different hospital levels. However, level A emergency departments used high grade PPE (P100/N100/FFP3 or powered air-purifying respirator) more often than emergency departments at level B and C hospitals. The implemented ICMs in different hospitals are shown in Table 3. Most of the hospitals were able to follow the guidelines of the DOH. However, in terms of additional ICMs, emergency departments of level C hospitals used more restrictive measures when transferring patients in and out. The use of ICMs in public and private hospital was significantly different in patients who were transferred out. The timing of PPE usage or implementation of ICMs is shown in Table 4. Eighty percent (80/100) of hospitals completely implemented use of PPE, and 66% (66/100) of hospitals implemented their ICMs at the late stage of the SARS outbreak. The instruction to use PPE originated from emergency department managers in 60% of level A, 46% of level B, and 23% of level C hospitals. The order to implement ICMs came from hospital managers in 33% of level A, 50% of level B, and 62% of level C hospitals.

**Table 2:** Supply of personal protection equipment (PPE) in emergency departments by hospital level and type

PPE	Hospital level				Hospital type		
	A (%), n = 15	B (%), n = 28	C (%), n = 57	p value	Public (%), n = 30	Private (%), n = 70	p value
Basic PPE							
Head or shoe covers	13 (87)	25 (89)	52 (91)	0.862	27 (90)	63 (90)	1.000
Goggles	12 (80)	21 (75)	46 (81)	0.529	27 (90)	52 (74)	0.182
Face shield	15 (100)	25 (89)	47 (82)	0.182	26(87)	61(87)	0.948
Gloves	14 (93)	24 (86)	55 (96)	0.187	27 (90)	66 (94)	0.425
Apron	11 (73)	22 (79)	42 (74)	0.876	22 (73)	53 (76)	0.805
Disposable gown	10 (67)	25 (89)	35 (61)	0.030	23 (77)	47 (67)	0.476
Surgical mask	10 (67)	17 (61)	38 (67)	0.855	21 (70)	44 (63)	0.493
N95 respiratory mask	12 (80)	23 (82)	56 (98)	0.014	26 (87)	65 (93)	0.322
High grade PPE							
P100/N100/FFP3	11 (73)	13 (46)	5 (9)	0.000	14 (47)	15 (21)	0.011
Powered air-purifying respirators	6 (40)	2 (7)	4 (7)	0.001	5 (17)	7 (12)	0.347

**Table 3:** Implemented infectious control measures in different hospitals by level and type*

Infection control measures (ICM)	Hospital level				Hospital type		
	A (%), n = 15	B (%), n = 28	C (%), n = 57	p value	Public (%), n = 30	Private (%), n = 70	p value
Basic ICM							
Entrance body temperature screen	15 (100)	28 (100)	57 (100)	-	30 (100)	70 (100)	-
Visitors restriction	14 (93)	24 (86)	55 (96)	0.193	30 (100)	63 (90)	0.347
Quarantine of fever patients outside of EDs	14 (93)	28 (100)	56 (98)	0.341	29 (97)	69 (99)	0.533
Quarantine fever patients in isolation room	15 (100)	24 (86)	46 (81)	0.158	26 (87)	59 (84)	0.760
Instituted fever screening station	15 (100)	28 (100)	55 (96)	0.847	30 (100)	68 (97)	0.373
Additional ICM							
Instituted fever triage ward	11 (73)	22 (79)	38 (67)	0.252	24 (80)	47 (67)	0.194
Restricted fever patient admission	13 (87)	21 (75)	52 (91)	0.128	26 (87)	60 (86)	0.900
Restricted patients transfer in	5 (33)	7 (25)	31 (54)	0.026	13 (43)	30 (43)	0.965
Suspected case-patients transfer out	4 (27)	9 (32)	43 (75)	0.000	12 (40)	44 (63)	0.048
SARS screening team	8 (53)	13 (87)	40 (70)	0.087	16 (53)	45 (64)	0.303
Closure of ED	3 (20)	5 (18)	15 (26)	0.654	9 (30)	14 (20)	0.276

*ED, emergency department; SARS, severe acute respiratory syndrome.

**Table 4:** Features of infectious control measures in different hospitals by level and type*

Response to the SARS outbreak	Hospital level				Hospital type		
	A (%), n = 15	B (%), n = 28	C (%), n = 57	p value	Public (%), n = 30	Private (%), n = 70	p value
Timing of PPE (complete preparedness)				0.132			0.082
Early stage	4 (27)	2 (7)	14 (25)		3 (10)	17 (24)	
Late stage	11 (73)	26 (93)	43 (75)		27 (90)	53 (76)	
Start of PPE use				0.015			0.006
From ED workers themselves	1 (7)	2 (8)	8 (14)		3 (10)	8 (11)	
From ED administrators	9 (60)	13 (46)	13 (23)		17 (57)	18 (26)	
From hospital administrators	5 (33)	13 (46)	36 (63)		10 (33)	44 (63)	
Timing of ICMs (complete set-up)				0.087			0.559
Early stage	3 (20)	8 (29)	24 (42)		10 (33)	24 (34)	
Late stage	12 (80)	20 (71)	33 (58)		20 (67)	46 (66)	
Order of infection measures				0.058			0.136
From ED workers themselves	1 (7)	1 (4)	7 (12)		3 (10)	7 (10)	
From ED administrators	9 (60)	13 (46)	15 (26)		16 (53)	21 (30)	
From hospital administrators	5 (33)	14 (50)	35 (62)		11 (37)	42 (60)	

*SARS, severe acute respiratory syndrome; PPE, personal protective equipment; ICM, infection control measures; ED, emergency department.

Table 5 shows the rating scales and correlations of difficulty and effectiveness of ICMs in emergency departments. Of the basic ICMs, instituting isolation at emergency departments was thought to be the most difficult (median = 3 score). Additional ICMs were more difficult to implement than basic ICMs. The effectiveness of additional ICMs was also thought to be less than that of basic ICMs. Closing emergency departments was thought to be less effective. Overall, the effectiveness of ICMs decreased as difficulty of implementation increased. Significant negative correlations were seen between perceived difficulty and effectiveness of implemented ICMs, except for body temperature at admission, institution of a fever screening station, and closure of the emergency department.

**Table 5:** Rating scale and correlation of difficulty and effectiveness of infection control measures (ICM) implemented*

Response to the SARS outbreak	Difficulty		Effectiveness			
	Median	IQR	Median	IQR	Correlation	p value
Basic ICM						
Entrance body temperature screen	1	1	5	1	–0.061	0.55
Visitors restriction	2	2	4	2	–0.309	0.002
Quarantine of fever patients outside of EDs	2	3	5	1	–0.283	0.005
Institution of isolation room at ED	3	3	4	1	–0.226	0.026
Institution of fever screening station	1	2	5	1	–0.128	0.214
Additional ICM						
Institution of fever triage ward	3	2	4	4	–0.210	0.042
Restriction of fever patient admission	3	2	4	2	–0.283	0.005
SARS screening team	4	2.25	4	3	–0.408	0.000
Restriction of transfer in	4	2	3	2	–0.269	0.010
Suspected cases transfer out	3	3	3	3	–0.210	0.040
Closure of ED	4	2	2	3	–0.153	0.140

*SARS, severe acute respiratory syndrome; IQR, interquartile range; ED, emergency department.

## Discussion

### Impact of SARS on Emergency Departments in Larger Hospitals

In our study, emergency department workers in larger hospitals were more severely affected by the SARS outbreak than staff at smaller hospitals. Several possible reasons could explain this finding. First, as our data showed, emergency departments at larger hospitals tend to have more patients requiring emergency services than those at smaller emergency departments. Thus, overcrowding and more frequent contact with patients would increase the incidence of person-to-person transmission. Emergency department workers may have become infected even without contact with a hospitalized SARS patient (14). Second, most larger hospitals were located in an urban area. Persons living in urban areas may be more likely to travel overseas, which would increase their chances of contracting an infectious disease. A similar phenomenon was reported at hospitals in cities with a high population density, such as Beijing, Hong Kong, Singapore, and Toronto (14–19). Third, fever patients tended to visit larger hospitals in the belief that they would be able to see a specialist who could identify the fever source. Finally, emergency departments at larger hospitals had more patients transferring in, so some patients with fever of unknown origin may have been transferred from lower level hospitals.

In Taiwan, emergency department volume does not always correlate well with either hospital bed number or with hospital location (such as urban versus. nonurban). This lack of correlation may explain, in part, the large variation in numbers of emergency department patients in level A hospitals. During the SARS outbreak, some patients were transferred to larger hospitals in both urban and semiurban areas. Therefore, preventing outbreaks at larger hospitals during an epidemic of an emerging disease is essential.

### Use of High-Grade PPE and Additional ICMs

In our analysis, most of the emergency departments in Taiwan followed the guidelines for basic PPE provided by the DOH. Some hospitals did not use these basic recommended PPE because they already had other PPE that performed the same function. However, some hospitals may have had an inadequate supply of PPE. In fact, a substantial problem for hospitals during the SARS epidemic was the cost of basic PPE, such as surgical masks and N95 respiratory masks, which increased costs markedly during this period. The quantity of PPE required by larger hospitals was very large, which placed a financial hardship on these hospitals, even though many of them did not encounter any SARS cases. Some hospitals were so anxious to acquire sufficient basic PPE that they even requested recycled PPE if it was available. The supply of higher grade respirators was greater at larger hospitals than at smaller hospitals. This finding may have been because hospital outbreaks were generally more common at larger hospitals and more patients who needed emergency resuscitation were transferred to larger hospitals. Transfers inevitably increased the risk for transmission to emergency workers (14).

In our analysis, most hospitals implemented basic ICMs during the SARS epidemic, but smaller emergency departments more frequently used more restrictive ICMs. Paradoxically, smaller hospital appeared to be more alert to the emerging disease, although the number of emergency department patients was lower than in larger emergency departments. This may have been because smaller hospitals were aware that they lacked the ability and capacity to treat SARS patients and therefore implemented additional ICMs to prevent an outbreak. Smaller hospitals were more likely than larger hospitals to restrict the patients from being transferred in than to transfer out suspected case-patients during the epidemic. Placing the suspected SARS patients in an isolation room was recommended. However, most hospitals, both public and private, found this a considerable challenge (20–22). Lack of isolation rooms became the key reason for transferring patients out and restricting the transfer of patients in. Because of the difficulty of isolating all suspected patients, implementing additional ICMs became the best strategy for most emergency departments. This strategy may have resulted in more patients with fever being transferred to a large hospital, thereby exposing these hospitals to a high risk of an outbreak. An inadequate number of isolation rooms will still be a problem in the next large-scale epidemic.

### Use of PPE and ICMs in Late Stage of Epidemic

Because the effects of SARS on the healthcare system were unknown in early stages, most hospitals had no clearly defined response plan and were unsure when to implement ICMs. In our analysis, use of PPE or ICMs in the emergency department usually began at the outset of the epidemic (outbreak at hospital A). The attitude of most hospital administrators was to keep an eye on the situation, especially in private hospitals. Administrators were concerned that additional ICMs would decrease the volume of services. This attitude was similar to that of health policymakers in the initial stage who were concerned that the SARS epidemic would have a devastating effect on the nation's economy and would cause widespread panic. Few hospitals actually prepared an infection response plan in the early stage that included the preparation of PPE and design of ICMs. Some hospitals did not even begin to consider how to implement these measures until they were directly facing the SARS epidemic.

PPE and ICMs were a financial hardship for private hospitals (>80% of hospitals in Taiwan). The reimbursement for private hospitals comes from the Bureau of National Health Insurance, depending on what services are provided. During the SARS epidemic, the overall volume of patients decreased, which affected the income of emergency departments and hospitals (23). In a future epidemic, without immediate government assistance at the crucial early stage, the effectiveness of hospitals' response will be reduced.

### Implementing Additional ICMs

Implementing basic ICMs was easier and more effective than implementing additional ICMs. Most of the basic ICMs were directly ordered by the DOH, so hospitals were required to fully support the emergency departments. This fact may explain why these basic ICMs were rated low in difficulty to implement and thought to have high effectiveness. Whether additional ICMs protected smaller hospitals is unclear, but these measures did appear to decrease the risk of an outbreak in lower level hospitals. However, difficulty and effectiveness of ICMs had a significant negative correlation. This finding may have been because physicians were required to spend more time communicating with patients or with outside hospitals, which also had the effect of causing the number of complaints and disagreements between physicians and patients to rise. The effectiveness of additional ICMs could be increased by making their implementation less difficult. Some of these additional ICMs will place a great strain on the healthcare system and render it incapable of functioning normally. Early recognition and rapid initiation of infection control precautions are the most important strategies for controlling large-scale infectious disease outbreaks (24). If recognizing a new or large-scale contagious infectious disease in the early stage is not possible, implementing additional ICMs in hospitals, especially smaller hospitals, may be unavoidable because the first priority for hospital managers is to prevent a hospital outbreak. To avoid disrupting the healthcare system when additional ICMs are implemented, the DOH should do its utmost to provide full financial support and other assistance. If equal support from the DOH for all hospitals is not practical, a centralized system for suspected patients may be considered as a strategy to reduce the severity and extent of an epidemic. This strategy may decrease the high incidence of person-to-person transmission in larger hospitals and may enhance the ability of smaller hospitals to treat patients with suspected cases. Implementing a centralized system of quarantine is controversial, however, because ethical issues are involved (25).

The response rate of the present study was low, so results may have been affected by nonresponse bias. The nonresponding emergency departments may have had less effective systems in place when they responded to the SARS epidemic. Thus, the degree of variability among emergency departments may have been underestimated. In addition, little seasonal variation in emergency department volume occurs in Taiwan, and any seasonal variation in the 3-month period was likely to be negligible.

The findings of this study suggest that policymakers should understand the different abilities of hospitals to respond to an epidemic. In addition, support and control measures should be implemented more effectively and made immediately available to all hospitals, whether public or private. Understanding the ability and capacity of different hospitals to respond to a contagious disease will enable policymakers to design effective infection control measures to safeguard the health of the nation.
